# Psychological Factors That Contribute to the Use of Video Consultations in Health Care: Systematic Review

**DOI:** 10.2196/54636

**Published:** 2024-12-11

**Authors:** Helen M Haydon, James A Fowler, Monica L Taylor, Anthony C Smith, Liam J Caffery

**Affiliations:** 1 Centre for Online Health The University of Queensland Woolloongabba Australia; 2 Centre for Health Services Research The University of Queensland Woolloongabba Australia; 3 School of Public Health Faculty of Medicine The University of Queensland Brisbane Australia; 4 Centre for Innovative Medical Technology University of Southern Denmark Odense Denmark

**Keywords:** telehealth, video consultations, psychological factors, health professionals, health services research, psychological, video, systematic review, review, barriers, engagement, health services, cognitive, consultations, psychological barrier

## Abstract

**Background:**

There are numerous benefits to delivering care via video consultations (VCs). Yet, the willingness of health care professionals (HCPs) to use video as a modality of care is one of the greatest barriers to its adoption. Decisions regarding whether to use video may be based on assumptions and concerns that are not necessarily borne of evidence. To effectively address psychological barriers to VC, it is essential to gain a better understanding of specific factors (eg, attitudes, beliefs, and emotions) that influence HCPs’ VC use.

**Objective:**

This study’s aim was to conduct a systematic literature review of psychological factors in HCPs that impair or promote VC use.

**Methods:**

Databases were searched in February 2023 for peer-reviewed primary research papers on VCs that discussed psychological factors of health professionals affecting the use of video to deliver health services. A psychological factor was defined as an intraindividual influence related to, or in reaction to, VC use—in this case, the individual being an HCP. Search terms included variations on “telehealth,” “clinician,” and psychological factors (eg, attitude and beliefs) in combination. Peer-reviewed papers of all methodological approaches were included if they were in an Australian setting and the full text was available in English. Studies where the main intervention was another digital health modality (eg, remote monitoring and telephone) were excluded. Studies were also excluded if they only reported on extrinsic factors (eg, environmental or economic). Information extracted included author, year, medical specialty, psychological component mentioned, explanation as to why the psychological factor was related to VC use, and exemplar quotes from the paper that correspond to a psychological component. Each extracted psychological factor was classified as a positive, negative, ambivalent, or neutral perspective on VC, and a thematic analysis then generated the factors and themes. Theories of behavior were considered and discussed to help frame the interaction between themes.

**Results:**

From 4592 studies, data were extracted from 90 peer-reviewed papers. Cognitive and emotional motivators and inhibitors, such as emotional responses, self-efficacy, attitudes, and perceived impact on the clinician as a professional, all interact to influence HCP engagement in VCs. These factors were complex and impacted upon one another. A cyclical relationship between these factors and intention to engage in VCs and actual use of VCs was found. These findings were used to form the psychological attributes of VC engagement (PAVE) model. Evidence suggests that HCPs fall within 4 key user categories based on the amount of cognitive and practical effort needed to deliver VCs.

**Conclusions:**

Although further research is needed to validate the current findings, this study provides opportunity for more targeted interventions that address psychological factors impeding effective use of VCs.

## Introduction

Telehealth can provide advantages over traditional care delivery, including greater access to care; increased convenience; less disruption to activities of daily living; and lower costs for the patient and, in some cases, the health care professionals (HCPs) [1,2]. Additionally, there are societal benefits and environmental benefits to delivering care by telehealth [3,4]. Furthermore, when practiced appropriately, telehealth is noninferior and, in some instances, superior to usual care [5]. Hence, it would seem logical that telehealth would be a mainstay of health care delivery. However, this is not the case [6].

Clinician barriers are acting as impediments to the widespread adoption of telehealth. The seminal paper by Wade et al [7] concluded that clinician willingness to use telehealth was the key factor of successful telehealth. Building on this work, further research has identified that avoidance or hesitancy with telehealth, specifically the use of video consultations (VCs), results from HCPs’ worries, assumptions, or preconceived frames of reference (eg, assuming the patient will not have access to technology) as opposed to evidence-based on empirical research [8,9]. These authors have proposed pragmatic strategies to redress adverse cognitive and emotional reactions to VC, thereby increasing VC use. We know from previous literature that HCPs have mixed feelings about VCs, and attitudes can be quite context specific [10,11]. Some feel it improves patient prognosis and successfully supports their decision-making, and some face challenges with physical exam capabilities and the ability to order further tests [10]. However, a lack of willingness when there are no physical or practical barriers infers psychological factors at play [7]. To effectively address these psychological hurdles, it is essential to comprehend the specific factors (eg, attitudes, beliefs, and emotions) that influence HCPs’ VC use.

To the best of our knowledge, there have been no studies that have synthesized the psychological factors that influence HCPs’ use of VC. Common theoretical frameworks used to explain technology adoption include the technology acceptance model (TAM), unified theory of acceptance and use of technology, and technology organization environment frameworks [12-14]. Although used in the field of health and well-being, these frameworks are not specific to HCPs or health care organizations, and they include external variables influencing adoption. Australia is one country where there has been particularly limited uptake of VCs in health care compared with other high-income nations. For example, in 2022, while large health services in the United States, such as the Veterans Health Administration, were using video for over 50% of their mental health visits [15], less than one-quarter of Medicare-funded mental health appointments were done by phone or video in Australia [16]. The aim of this study was to specifically identify psychological factors in HCPs in Australia that impair or promote the use of VCs.

## Methods

### Operational Definition of a Psychological Factor

Psychological factors were defined as intraindividual influences related to, or in reaction to, VC use. We considered a range of psychological definitions, theories, and frameworks and scoped the literature for factors related to concepts such as cognitions, attitudes, beliefs, self-efficacy, intentions to use, motivation, preferences, and emotions.

### Search Strategy and Eligibility Criteria

This review protocol was not registered. Searches were run in the Embase, MEDLINE, CINAHL, and PsycINFO databases from inception to February 2, 2023. Inclusion and exclusion criteria were based on our predefined PICO (population, intervention, context, outcome) framework. Search terms included variations on “telehealth” (virtual and video), “clinician” (nurse and physician), and psychological factors (attitude and beliefs) combined. Peer-reviewed papers of all methodological approaches were included if they were in an Australian setting and the full text was available in English. Other inclusion factors were that the intervention involved synchronous video appointments between a patient and HCPs, and that the outcomes reported intrapersonal or intrinsic psychological factors that related to an HCP’s use of VC. The full search strategy for each database is included in Multimedia Appendix 1.

Studies where the main intervention was another digital health modality such as remote monitoring or phone appointments were excluded. Studies were also excluded if they only reported on extrinsic factors such as environmental or economic factors affecting HCPs use of VC. If the results of a study did not clearly differentiate between HCPs and patient factors, or between video and other modalities of care, it was excluded.

### Screening and Data Extraction

Search results were uploaded into Covidence software (Veritas Health Innovation) to perform the screening stage. Each paper’s title and abstract were screened by 2 reviewers independently and excluded if they did not meet the criteria. The full text of the remaining papers was also screened by 2 reviewers, resulting in a final list of included studies. Disagreements between reviewers (eg, whether the included factors were psychological in nature) were resolved by the remainder of the research team at regular meetings. Given the high volume of papers and in-depth level of extracting required, the included full-text papers were divided between authors for manual data extraction. First, a random sample of 5 papers was chosen for data extraction and completed by all reviewers independently. Results were then compared and checked to ensure consistency in the process. The remaining papers were then divided across reviewers with regular meetings to check for consistency and discuss any anomalies. Information extracted included author, year, medical specialty, psychological component mentioned, explanation as to why the psychological factor was related to VC use, and exemplar quotes from the paper that correspond to a psychological component. An example of a psychological component could have been “positive attitude toward using video consultations as they are patient centred.” One paper may have multiple psychological factors mentioned. Notes were also made if papers reported how much VC experience staff had, and whether staff underwent specific VC training.

### Data Analysis

For each psychological factor extracted, it was noted whether it represented a positive, negative, ambivalent, or neutral perspective on VC. Psychological component data points were then sorted according to this classification. Common themes across papers were categorized and summarized descriptively. A separate study team member was responsible for each classification. Primarily, the thematic analysis, which followed Braun and Clarke’s [17] approach, was inductive, with constant comparison of journal paper text and several peer debriefing meetings, resulting in the themes generated [18] and the proposed relationships between them. In the final stage analysis, during these peer debriefing meetings, theories of behavior were considered and discussed to help frame the interaction between themes and further explore patterns in the findings. Several theories were discussed including the theory of planned behavior (TPB) [19], the stages of change [20], and the normalization process theory [21] that potentially aligned with the inductive findings. Reporting followed the PRISMA (Preferred Reporting Items for Systematic Reviews and Meta-Analyses) 2020 guidelines [22].

### Risk of Bias Assessment

Each included paper was critically appraised using the Mixed Methods Appraisal Tool [23]. This checklist was selected as it can be used for most study designs, and we expected multiple study types to be included in our review. It was specifically developed for use during the study assessment stage of systematic reviews and has been evaluated and validated. The rating outcomes were examined by criterion and results were described descriptively as calculating an overall score is not advised by the tool creators.

## Results

### Summary of Included Studies

**Figure 1:**
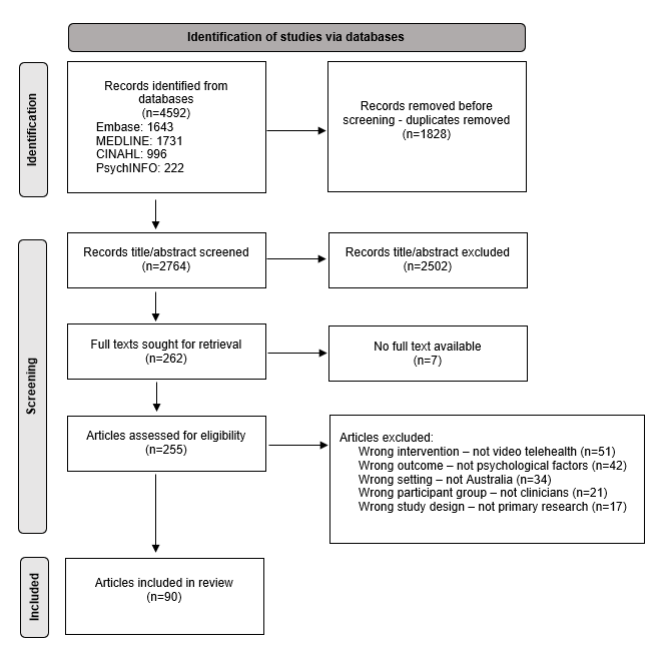
PRISMA (Preferred Reporting Items for Systematic Reviews and Meta-Analyses) diagram of the screening process.

An initial yield of 4592 studies was refined into 90 included papers [7,24-112]. Figure 1 contains an overview of the search and screening processes. Studies were published between 2001 and 2023, with more than two-thirds (62/90, 69%) being published in the last 3 years. The papers were most often focused on allied health topics including mental health (n=11), speech-language pathology (n=8), physiotherapy (n=6), and overall rehabilitation (n=7). There were also many studies that included a variety of HCPs across different contexts and disciplines (n=12). Staff positions varied both within and between studies, such as nurses, general practitioners, and specialists. The study designs included 43 qualitative studies, 34 mixed methods studies, 8 nonrandomized controlled trials, 4 quantitative descriptive studies, and 1 randomized controlled trial.

### The Psychological Attributes of VC Engagement

**Figure 2:**
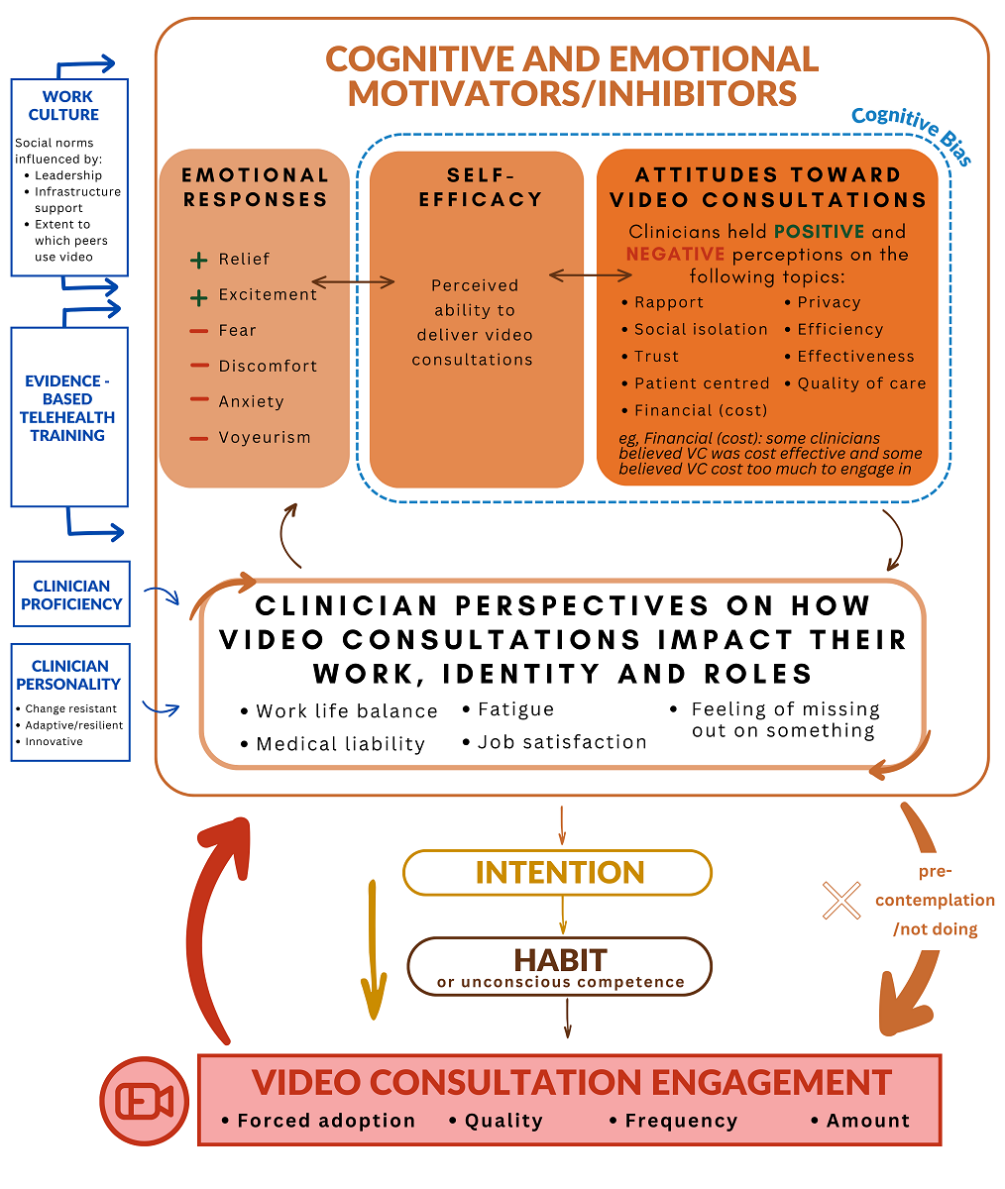
The psychological attributes of video consultation engagement (PAVE) model showing interacting cognitive and emotional contributors to engaging in video consultations and their perceived impacts on the clinician. Examples included (eg, emotions and attitudes) in this diagram represent the most dominant themes and common examples from the systematic review and do not account for all that was found.

Cognitions refer to mental processes involved with memory, attention, problem-solving, and perceptions, whereas emotions refer to feelings or affective states [113]. Literature suggests that the constructs of emotion and cognition (eg, affective attitudes) are highly related but can be considered independent factors [113,114]. Unsurprisingly, our analysis found that cognitive and emotional motivators or inhibitors to engage in VCs were complex, overlapping, and impacting upon one another. Using the findings from this study, we developed the psychological attributes of video consultation engagement (PAVE) model (Figure 2), which highlights the cyclical relationship between cognitive and emotional contributors (including those pertaining to clinician identity and professional role) and engaging in VCs. Intention to use VCs and its relationship with habit and the actual behavior of engaging in VCs are also presented. It should be noted that examples included in the diagram represent the most dominant themes and what was most common in the systematic review but do not necessarily account for all that was found.

The following sections describe the psychological factors and themes that were generated, but it must be emphasized that these constructs are interactive and related. Further, a central psychological factor (eg, attitudes) could be both a motivator (pro-VC) or inhibitor (anti-VC). For instance, fear (anxiety) of doing VCs is influenced by one’s attitudes toward engaging in that behavior, which can, in turn, reinforce negative perceptions of VCs and diminish confidence to deliver VCs. Please note that when describing the psychological factors present in the literature, we do not critically examine or cross-reference evidence to support or refute such perspectives.

### Cognitive and Emotional Motivators or Inhibitors

Four main themes or intraindividual factors embody the majority of the literature results: emotional responses; self-efficacy; attitudes toward VCs; and subjective impact on clinician—role and identity (top rectangle in PAVE model). As suggested by the title, these factors can either motivate or inhibit the use of VCs or the intention to use video.

### Emotional Responses

Both positive and negative emotional responses to using VCs were expressed by HCPs across the literature. HCPs’ fear, anxiety, and discomfort were often mentioned or alluded to, across the research [47,53,56,71,79,85,87,91,95-97, 99,101-104,115]. Conversely, excitement and relief were also responses to doing VCs [49,86]. The cyclical nature was evident when HCPs initially found it uncomfortable to do VCs but after persisting, they were relieved that it was something they no longer needed to think about (reduced cognitive effort) [55,73,78,87]. An unexpected finding was the sense of voyeurism that HCPs sometimes reported in the literature [56,78]. For example, HCPs uncomfortable “watching” a patient through a screen as they reveal sensitive information or body parts (eg, genitals) [56]. Feelings of voyeurism were also reported when HCPs felt like they were invading a patient’s privacy, due to seeing into their living quarter [78].

### Attitudes Toward VCs

Attitudes refer to positive or negative personal evaluations of providing care via video [19]. As seen in Figure 2, a range of attitudes toward VCs were found across the literature mostly pertaining to the 9 topics listed in the figure. Of note, HCPs’ attitudes, often about the same topic, were polarized and not necessarily evidence based. The following examples highlight the disparity in findings but do not include the full range of attitudes identified within the literature. Some HCPs believe you cannot build rapport with consumers via VCs (which is contrary to consumer perceptions) [26,29,35,51,54,70,73,76,78, 81,83,84,86,106,107,111,112,116], while other HCPs perceive VCs as being beneficial to rapport-building [61,87,91,92,97,107]. Some HCPs believe VCs increase social and professional isolation [32,51,53,70,74,84,115], while others perceive it to decrease isolation and increase clinical collaboration [24,59,61,93,95,97,98,102,110,117]. Some HCPs view VCs as a barrier to health care for consumers [26,47,58,93,99,103,104,106,108,111], while others perceive VCs as patient centered and promoting health care access [26,40,46,48,49,51,52,54,55,60,73,78,81,84,92,96,97,101,107]. Some described an attitude that telehealth would reduce the quality of care provided to patients, would be disruptive, or is inferior to physical care [26,33,35,36,48,52,56,58,60,63,70,71, 75,76,80-82,85,86,93,96-98,103,104,107,108,111,112,118], whereas others felt it would improve the quality of care provided and the lives of patients [7,26,33,45,51,77,78,84,85,87, 92,100,101,107,110-112]. As described by Sutherland et al [99], attitudes can be dependent on the context of use, with their findings showing HCPs were comfortable using video broadly, but not for completing assessments.

### Self-Efficacy

Self-efficacy was identified as a pivotal construct that interacted with a range of attitudes toward and emotional responses from engaging in or delivering VCs. We define self-efficacy as HCPs’ beliefs that they could provide high-quality telehealth care and overcome technological challenges that may inhibit this [119]. Many HCPs remarked that they did not feel confident in delivering VCs [53,56,79,84,87,95,96,98,101,102,104,107, 112,115]. For example, Pitt et al [87] described participants as being unable to manage technology or hardware problems if they were to emerge. However, persevering through barriers to providing VCs (such as some of the emotional factors described above) can build self-efficacy and evoke a positive emotional response [73,78,85,87,115]. For example, one HCP described at the beginning using video seemed “impossible” (low self-efficacy), but over time they became so equipped in its use they “no longer think about it” (high self-efficacy) [78]. Other research has shown that positive attitudes towards telehealth can also predict whether HCPs were willing to build self-efficacy to problem-solve technology failures [40].

### Cognitive Bias

Considering the interaction between these themes and the way in which decisions to use VCs are not always based on evidence but upon assumptions, cognitive bias is illustrated as a filter around the cognitive motivators and inhibitors. Cognitive biases are described as simplified information-processing strategies that may cause individuals to form incorrect perspectives [120]. A previous review has highlighted that within medical contexts, HCPs may be influenced by biases such as confirmation bias (intentionally selecting information that meets prior attitudes), or the bandwagon bias (a tendency to believe what the majority believe) [121]. Recent work by Cook et al [9] (published outside of this review period) has highlighted that many HCPs felt telehealth to be clinically inappropriate and not meeting consumer demands, despite evidence suggesting the contrary. This suggests that cognitive biases around telehealth being inappropriate were subconsciously driving telehealth uptake, rather than empirical evidence. Therefore, while the role of cognitive biases was not explored within the scope of the included papers, broader psychological theory highlights the role that they may have in VC uptake, particularly in driving the polarized attitude towards which appears disconnected from available evidence.

### How Clinicians Perceive VCs Impact Them Directly, Their Identity, and Roles

The final theme within the cognitive and emotional factors is the perceived impacts on the clinician, their identity, and professional roles. Although the elements within this theme can be considered cognitions or emotions, there was sufficient evidence within the literature to warrant a theme that was specifically related to the profession of being a clinician. While other factors were paradoxical, generally factors related to clinician impact were negative. For example, there was some evidence suggesting that clinicians feared that telehealth would take away their independence, the need for their roles, and broadly modify models of care to make clinicians less important [33,98,104,107,108,110,112,117]. There was evidence that some clinicians perceived VCs as increasing their workload [29,49,62,70,73,75,79,95,97,99,101,103,104,108,110,111]. However, some participants considered this positive, as workload increase stemmed from increased patient loads [59]. VCs also increase fatigue or cognitive load [35,73,104,106,111,116] and medical liability [75,107]. Some also expressed loss of job fulfillment because of videoconferencing [26,112,115], such as a genetic counselor who no longer had the opportunity to deliver good news as this component was reassigned to a different clinician within their model of care [112]. Finally, there are mixed results regarding job satisfaction. Some studies found VCs increased job satisfaction and a sense of achievement [52,59,98], and others reported decreased job satisfaction [104,112,115].

### Intention and Habit

Intentions, from a TPB vantage point, are direct antecedents to behavior and are influenced by an individual’s perceptions of social pressures, perceived control, and attitudes toward providing VCs [19]. Numerous studies assessed intention [28,31,72,92,93,101]. Findings were mixed regarding the reliability of intentions to predict actual engagement (behavior) in delivering VCs. For example, participants in the study by Swales et al [101] found that despite high interest in using VC, few provided VC services. Another study by Brunelli et al [34], which explored the closely related construct of attitudes, found similar results. As such, we drew upon TPB models that included habits that could account for such results. The role of habit is evident when HCPs revert to traditional and practiced behaviors when providing care via telephone to avoid increased emotional or cognitive effort. For example, in the study by Moffatt and Eley [79], many HCPs reported using phone calls rather than videoconferencing because they were quicker and required less cognitive effort to learn. Other participants in this study described having preferences for the “traditional approach” because they lacked the confidence (emotional effort) to learn videoconferencing.

### Engaging in VCs

Overall, the cognitive and emotional factors outlined above seem to have a direct impact on whether HCPs use video conferencing to provide consumers with health care that would otherwise be provided in person. They also have an indirect impact (as described above) through intentions. That is, research showed 4 main categories, and potentially 4 main “user categories” (Table 1) as follows: first, HCPs influenced by their attitudes (often negative), self-efficacy (low), and emotions (negative) do not seriously contemplate (are precontemplative) delivering care via video and therefore do not engage [33,79,102]. For example, participants in the study by Moffatt and Eley [79] described “just not wanting to learn technology.” Second, HCPs who exert cognitive effort and plan and think about doing VCs (intentions) but for varied reasons do not engage in VCs [71,111]. For example, participants in the study by White et al [111] described how VCs require “preparation.” Third, HCPs who exert cognitive effort and plan and think about doing VCs (intentions) before engaging in VCs. These HCPs are at a crossroads. If they use video so infrequently [36,111] like many skilled behaviors, the effort and planning remain because it is not practiced, and there is the potential for the behavior to stop. For example, White et al [111] described HCPs exerting effort to plan and deliver videoconferencing, but after ongoing technology issues they “abandoned” the format altogether. However, if HCPs have good experiences and engage in VCs regularly, they can progress to type 4. Fourth, HCPs who are so practiced and confident at delivering VCs that it takes minimal cognitive and practical effort (eg, high self-efficacy and effective supporting infrastructure) and has become business as usual (unconscious competence or habit) [49,52,55,78]. For example, Gelber [55] described participants as saying telehealth is “now second nature.”

**Table 1:** Four user categories based on the amount of cognitive and practical effort needed to deliver video consultations.

	Low/no effort (eg, cognitive and practical)	High effort (eg, cognitive and practical)
Not engaged	Type 1. Characterized by one or more of the following: negative attitudes, low self-efficacy, and negative emotional reactions to use Precontemplation: No engagement	Type 2. Cognitive effort and thinking about doing video consultations (intentions) but does not translate to behavior sometimes due to infrequent use and loss of skill and confidence No engagement or limited or infrequent use means reverting to habit eventuating in extinguished behavior
Engaged	Type 4. Practiced and confident clinicians push through barriers with minimal cognitive and practical effort (eg, high self-efficacy and effective supporting infrastructure) Business as usual or unconscious competence	Type 3. Cognitive effort and think about doing video consultations (intentions) before engaging in video consultations Engage or persist

The extent to which clinicians engage in VCs can impact the emotional responses, self-efficacy, attitudes, and perceived professional impact. As described by Ayres et al [26], practitioners struggled due to their variations of experiences and an inability to “predict” the next consultation outcome. Specifically, the amount and frequency of engaging in VCs, as shown by studies where HCPs were initially reluctant, but after use, continued to use it more [85,87,94,102,109]. Technology factors, such as the quality of the technology, can create frustration and contribute to technology anxiety or fears [49,55,56,71,100,104,111]. For example, one study reported that audiovisual interruptions can make practitioners “anxious” [53]. However, these can also be influenced by self-efficacy and how confident HCPs were to work with technology issues [7,73]. For example, Lawson et al [73] described technology issues as a way to “break the ice”, and Wade et al [7] described how HCPs may accept VC despite technology issues. The audiovisual quality of VCs is important if HCPs are anxious about appearing incompetent to their patient [56,102]. Forced adoption or pressure to use video can lead to resentment toward VCs [102-104]. Similarly, the rapid uptake of telehealth as part of COVID-19 social distancing requirements [6] meant that HCPs were unprepared, untrained, and unsupported, which led to negative responses and low self-efficacy [71,90,91,101,102,104]. In comparison, the “forced adoption” as a result of COVID-19, also exposed HCPs to the benefits of VCs and led to acceptance and more regular use [102].

### Other Factors Impacting the Cognitive and Emotional Motivators or Inhibitors

Finally, several factors were found in the literature that are important to consider and overlap or influence the cognitive and emotional factors. Work culture and the social norms influenced by leadership, infrastructure support, and peer influence can influence attitudes toward VCs and self-efficacy [70,76,85,91,102,103]. Similarly, adequate evidence-based VC training can increase self-efficacy and elevate attitudes toward VC and emotional responses [70,86]. More stable factors, like personality, are represented by research that discusses HCPs being change-resistant or adaptive and innovative [26,61,66,86,102,111], all of which may impact how a person responds to or views VCs.

### Quality Assessment Results

Overall, there was good methodological quality in the included studies. The majority (50/90, 56%) were positively rated on all appraisal categories for their respective study types. For qualitative studies, there was not always coherence between the data collected, analysis, and interpretation. Coding processes and qualitative theories could have been expanded upon in more detail. In many mixed methods studies (18/34, 53%), the quantitative and qualitative data were not actually integrated, so it was not clear what the benefit was of a mixed methods study over just 2 separate studies. Nonrandomized studies often did not clearly discuss confounders. Full quality assessment rating results are available in Multimedia Appendix 2.

## Discussion

### Overall Findings

The aim of this systematic review was to identify HCP psychological factors that influence the use of VC for the delivery of health care. Research literature describes a range of complex and interacting psychological factors that influence whether HCPs engage in VCs. Cognitive and emotional motivators and inhibitors, such as emotional responses, self-efficacy, attitudes, and perceived impact on the clinician as a professional, all interact to influence HCP engagement in VCs. In turn, experiences of engaging in VCs impact and shape the cognitive and emotional motivators and inhibitors. Potentially mediating this cyclical relationship are intention and habit.

### Managing Attitudes and Emotions, and Other Technology Acceptance Literature

Attitudes, self-efficacy, and intention to use have been considered in other reviews [122] and are central to established models of technology acceptance, such as the TAM [123-125]. The TAM is based on the TPB [19], and like the underpinning behavioral theory, has been modified to examine potential psychological contributors to behavior [126]. For instance, extensions have included an examination of technology-related self-efficacy and its relationship to attitudes and intention to use a technology [127,128]. Interventions and training to increase knowledge and self-efficacy and ultimately VC use have been developed and have contributed to some increases in use [129]. The factor or theme we label as attitudes toward VCs, especially when considering the extent to which VCs are perceived to be effective or efficient, seems to overlap with the TAM construct of perceived usefulness. The research literature presents a range of HCP attitudes toward VCs and how these may be barriers or motivators to do VCs. Attitudes are predominantly polarized being for or against VCs. The lack of consistency across attitudes highlights their complexity and interaction with other factors. For instance, HCPs were divided on whether or not the use of VCs: could reduce or increase social isolation; was patient centered or prohibitive to patients; saved money or was costly; was effective or efficient; was trustworthy; increased or decreased privacy; and reduced or improved quality of care. Finally, there was a range of perspectives regarding the ability to build rapport via video. Regardless of direction, attitudes toward VCs impact and are informed by emotional responses and self-efficacy, which then impact the intention to use VCs or the actual use.

Although there is varied terminology and conceptualizations of emotions (eg, as affective attitudes), the bidirectional interplay between attitude and emotion is well established [130]. Emotions are integral in the processing of information and the formation of attitudes [131,132] and, in turn, attitudes impact emotional responses [133]. Specifically, there is a large body of work that demonstrates the relationship between emotions, especially technology anxiety, and its impact on willingness to engage in technology-related activities such as VCs [79,86,134,135]. Exposure therapy is one way to mitigate HCP anxiety [136], but simply repeated positive experiences and support engaging with VCs can reduce discomfort and lead to relief and sometimes even excitement [49,86,87].

### Addressing Health Professional Confidence and Identity

Overlapping with attitudes, emotions, and self-efficacy, there were factors that focused specifically on roles and identity as an HCP. There were numerous papers mentioning fears that telehealth would reduce professional independence and make their roles redundant. Concerns also extend to feelings they could not properly control the patient or the consultation, which influences their willingness to engage in telehealth and the perceived usefulness of health technologies [137]. Therefore, to facilitate future VC uptake, it is important to use appropriate training and education. Training should upskill HCPs to improve their ability to conduct and control VCs effectively. Education should relay the vast evidence demonstrating that technology does not necessarily lead to skill reduction and job loss, but rather, it is most likely to augment workforce capability, freeing up HCPs to conduct advanced scope work [138]. Most importantly, mitigating negative impacts on workload and professional roles can be achieved through comprehensive implementation strategies such as amending workflows and increasing administration time.

### Changing HCPs’ Biases

Our findings suggest that the HCP cognitions and emotions are filtered through cognitive biases. As described by Cook et al [9], how a clinician perceives telehealth is a key driver of whether telehealth is provided regardless of any empirical evidence supporting telehealth use that may be present. Decisions regarding whether or not to offer VCs, are shrouded in assumptions. All humans make decisions based on simplified information processing called heuristics, which often leads to inaccurate judgments and systematic assumptions called cognitive biases [139,140]. The role of cognitive bias in technology-related decision-making is emerging [139]. Although there are numerous types of cognitive biases, Oschinsky et al [139] examines the status-quo bias. The status-quo bias explains people’s tendency to maintain the “status-quo” or existing behaviors [141]. The potential of status-quo bias to increase resistance to technology use is evident [139,141]. The role of cognitive bias was not explicitly mentioned within the systematic review data but provided an explanation of the varying attitudes across the literature and relationships across psychological factors. However, further research is needed to examine which types of cognitive biases [142,143] may be impacting HCPs’ decisions to use VCs and where cognitive biases may be present. One possible example pertains to the cognitive bias called confirmation bias (a tendency to favor information that aligns with personal values or perceptions). Confirmation bias is at play when an HCP has one bad experience with audio on a call, which confirms their belief that VCs are inefficient and provide low-quality care. In contrast, an HCP with a positive attitude toward VCs will minimize the impact of a bad audio experience. A recent review [144] indicates that key strategies to change HCP cognitive biases are reflection and education. However, how to integrate this training in VC contexts specifically warrants further research. Approaches that target cognitive and emotional factors alongside cognitive biases may provide an optimal structure for behavior change.

### The “Intention-Behaviour Gap” and Unconscious Competence

Our PAVE model suggests that a range of emotions and cognitions can influence the intention to engage in VCs, but intentions do not always predict actual engagement [145,146]. For example, a previous review [147] suggests that intentions explain between 18% and 23% of the variance in actual behavior engagement. The inconsistent relationship between intention and actual behavior, termed the “intention-behaviour gap,” has long been recognized and examined across multiple behaviors [148,149]. Variations to the TPB and testing of moderating effects continue. One such moderator when considering the relationship between intention to engage in VCs and actual engaging in the behavior of conducting VCs, is habit.

Research indicates that when an individual frequently engages in a behavior, the predictive value of intentions, attitudes, and self-efficacy diminishes [150]. This is because frequently engaging in behavior forms a habit and reduces the reliance on cognitive effort to elicit the behavior and it becomes automatic. Evidence within our literature review indicated that, over time, with frequent use, many HCPs no longer consciously thought about how to use VC, because it had become automatic. “Unconscious competence” is cited frequently within knowledge acquisition studies [151] and is consistent with the findings within the current review. Conversely, influenced by attitudes, self-efficacy and other emotional reactions to VCs, the cognitive and practical effort needed to make VCs business as usual can be overridden by habit. If experiences are negative or the use of VCs is infrequent, there is the potential to “fall back into old habits” of using phone or traditional in-person care. Examining information technology use, de Guinea and Markus [152] argue that habit may moderate the relationship between intention and behavior, especially when repeated IT use (behavior) becomes habitual and so the need for the cognitive effort associated with intention diminishes. Research dating back to 2009 has not found conclusive evidence and, as such, there is a need to further examine the role of habit among other psychological factors.

### User Categories and Targeting Interventions

Finally, our findings suggest that HCPs may fit into 4 different user categories (Table 1). These include (1) individuals with negative cognitions and emotions and, therefore, do not seriously contemplate engaging in VCs; (2) those who exert cognitive effort and have intentions but for varied reasons do not progress to actually carrying out any VCs; (3) those who intend to use VC and exert cognitive effort to plan VCs, before engaging with VCs. This is a turning point. If the HCP does not regularly engage or has a bad experience, it could produce negative emotions and attitudes and the HCP stops using video. Alternatively, they may progress to the type (4) who use VC regularly and VC becomes automatic or habitual.

**Figure 3:**
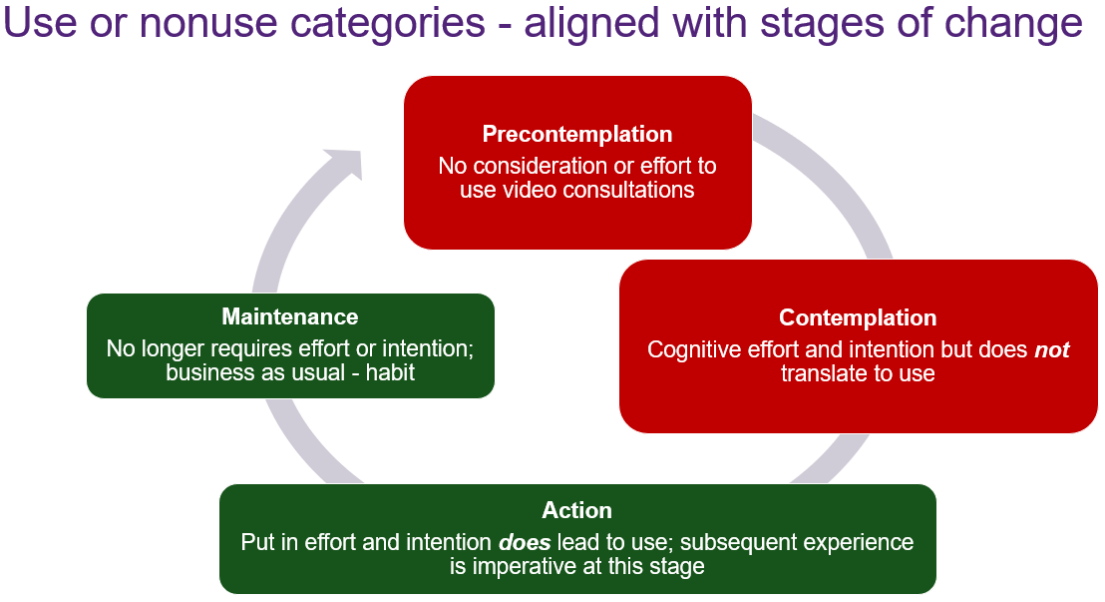
Use or nonuse categories as they align with The Stages of Change Model.

Although further examination of these 4 user categories is needed, it may also be prudent to examine them in alignment with the stages of change model (Figure 3) [153]. If future research supports the categorization of these 4 types of alignment with the stages of change model, targeted interventions based on this model could be developed. The stages of change model, traditionally based on addictive behaviors, is aligned with a range of interventions dependent on the stage of change. For instance, different interventions would be needed for someone who is not even contemplating using VC (eg, motivational interviewing exploring concerns and promoting the benefits) compared with someone who has progressed to using it once or twice (increase skills and self-efficacy through practice). One complexity that may counter the alignment with stages of change is that the choice and use of VCs may be context dependent, with providers showing different levels of enthusiasm depending on the clinical requirement.

### Strengths and Limitations

The authorship team has worked for considerable years within telehealth contexts, including directly with HCPs, which strengthens the data analysis. A limitation is the focus of the literature predominantly on intentions and not actual behavior. As described above, the influence of intention on behavior is not always consistent. The inductive analysis and interpretation of the relationship between psychological factors and the 4 user categories need to be further examined. In addition, individual study quality as detailed in the risk of bias assessment section may affect the validity of findings. It is also a limitation that due to the number of included papers our research team only had the capacity for 1 reviewer to initially code the data. However, a sample of 5 papers was analyzed by all researchers to improve interrater reliability, a second researcher was involved in collating the themes by classification (positive, negative, neutral, ambivalent), a third researcher reviewed overall themes, and regular peer debriefing meetings occurred with all researchers attending.

Heterogeneity in terms of clinical specialties and settings was both a strength in terms of generalizability, but also a limitation in aggregation of data, potentially increasing bias from studies. Profession was not always clearly identified in the journal papers; for instance, some studies simply stated that “health professionals” were interviewed. Further research focusing on individual clinician specialties (eg, do allied health professionals have more motivating attitudes?) could also increase understanding of this topic. However, 5 reviewers and the implementation of rigorous methods (eg, multiple peer meetings) instill confidence in the findings, especially as these factors or themes are widespread due to the shared experience reflected in human behavior. Finally, although the current aim and scope of this research were focused on Australia, it is expected that these results are somewhat generalizable beyond Australia.

### Conclusion

This review identified a range of factors that interact to influence the use of VCs. Cognitive and emotional factors that motivate (positive perceptions) or inhibit (negative perceptions) engagement in VCs were found. These include but are not limited to emotional responses (eg, feeling relief or anxiety), varying attitudes toward rapport-building, trust, and patient-centeredness, as well as effectiveness and quality of care. The impact of VC use on an HCP’s professional role and identity was also mentioned throughout the literature analyzed, alongside their perceived ability or self-efficacy. Our PAVE model (Figure 2) highlights the potential cyclical nature and relationships between the cognitive and emotional factors and intention to use or engage in VCs. The PAVE model highlights psychological factors and the relationships between them, which may be important when developing strategies that support clinicians in the use of VCs. Finally, HCPs may fall within 4 key user categories, which can help with targeting solutions when there is low VC uptake. These categories are as follows: (1) negative cognitions and emotions and no VC contemplation; (2) cognitive effort and intentions but do not progress to VC use; (3) planning and engaging with VC; and (4) use VC regularly, automatically or habitually. Further research validating the findings of this review can lead to interventions such as training, education, and reflective practices that address these psychological factors with the aim of delivering care via video when it is clinically appropriate.

## Appendix

Multimedia Appendix 1 Database search strategies.

Multimedia Appendix 2 Quality assessment of included studies.

Multimedia Appendix 3 PRISMA (Preferred Reporting Items for Systematic Reviews and Meta-Analyses) 2020 checklist.

## Abbreviations

HCP: health care professional
PAVE: psychological attributes of video consultation engagement
PICO: population, intervention, context, outcome
PRISMA: Preferred Reporting Items for Systematic Reviews and Meta-Analyses
TAM: technology acceptance model
TPB: theory of planned behavior
VC: video consultation

## Data Availability

The datasets generated and analyzed during this study are available from the corresponding author on reasonable request.
