# Supplementary material for: Psychological Factors That Contribute to the Use of Video Consultations in Health Care: Systematic Review
Source: J Med Internet Res. 2024 Dec 11;26:e54636. doi: 10.2196/54636 (PMC11670263; doi:10.2196/54636)
Supplement: Multimedia Appendix 1 [file jmir_v26i1e54636_app1.pdf]

Search strategies

“Telehealth adoption & the psychological factors that contribute to the use of video consultations in healthcare”

Embase:

No.	Query
#23	#22 AND ('article'/it OR 'article in press'/it OR 'review'/it) AND [english]/lim
#22	#6 AND #10 AND #18 AND #21
#21	#19 OR #20
#20	'australia'/exp OR 'australian'/exp
#19	australia* OR queensland* OR 'new south wales' OR 'northern territory' OR tasmania OR 'australian capital territory' OR victoria$ OR 'western australia' OR 'south australia'
#18	#11 OR #12 OR #13 OR #14 OR #15 OR #16 OR #17
#17	'health personnel attitude'/exp OR 'theory of planned behavior'/exp OR 'cognitivism'/exp OR 'behaviorism'/exp OR 'decision making'/exp OR 'thinking'/exp OR 'employee attitude'/exp OR 'attitude to computers'/exp
#16	
#15	((patient$ OR client$) NEAR/2 cent*d):ti,ab
#14	((past OR previous) NEAR/2 (experience$ OR behavi$r*)):ti,ab
#13	emotion*:ti,ab OR confidence:ti,ab OR confident*:ti,ab OR enjoy*:ti,ab OR fear*:ti,ab OR feel*:ti,ab OR identity:ti,ab OR identities:ti,ab OR inertia:ti,ab OR intent*:ti,ab OR interest$:ti,ab OR intrapersonal:ti,ab OR intrinsic:ti,ab OR motivat*:ti,ab OR opinion$:ti,ab
#12	(decision$ NEAR/2 (making OR made OR make$)):ti,ab
#11	affect*:ti,ab OR anxiety:ti,ab OR anxieties:ti,ab OR attitude$:ti,ab OR aversion$:ti,ab OR belief$:ti,ab OR bias:ti,ab OR cognitive:ti,ab OR concern$:ti,ab OR behavio$r*:ti,ab
#10	#7 OR #8 OR #9
#9	'health care personnel'/exp
#8	((healthcare$ OR care$ OR health$ OR general OR clinic$ OR hospital$) NEAR/4 (nurse$ OR nursing$ OR profession* OR practitioner$ OR clinician$ OR personnel$ OR worker$ OR provider$ OR staff* OR doctor$)):ti,ab
#7	physician$:ti,ab OR nurse*:ti,ab OR nursing:ti,ab OR 'health care professional':ti,ab OR 'health care personnel':ti,ab OR 'healthcare worker$':ti,ab OR 'healthcare personnel':ti,ab OR practitioner$:ti,ab OR doctor$:ti,ab OR clinician$:ti,ab OR provider$:ti,ab
#6	#1 OR #2 OR #3 OR #4 OR #5
#5	'videoconferencing'/exp OR 'telehealth'/exp OR 'teleconsultation'/exp
#4	((consult* OR care) NEAR/2 (virtual* OR 'e health' OR ehealth OR remote OR video$ OR electronic$)):ti,ab
#3	(tele$ NEAR/2 (medicine OR nursing OR health OR consult* OR care OR intervention$)):ti,ab
#2	((video NEAR/1 conferenc*):ti,ab) OR 'video*conferenc*':ti,ab
#1	'tele*health':ti,ab,jt OR 'tele*medicine':ti,ab,jt OR 'tele*nursing':ti,ab,jt OR 'tele*care':ti,ab,jt OR 'tele*consult*':ti,ab OR 'tele*intervention$':ti,ab

PsycINFO:

((TI ("tele*health" OR "tele*medicine" OR "tele*nursing" OR "tele*care" OR "tele*consult*" OR "tele*intervention#") OR AB ("tele*health" OR "tele*medicine" OR "tele*nursing" OR "tele*care" OR "tele*consult*" OR "tele*intervention#") OR SO ("tele*health" OR "tele*medicine" OR "tele*nursing" OR "tele*care")) OR (TI ((video N1 conferenc*) OR "video*conferenc*") OR AB ((video N1 conferenc*) OR "video*conferenc*")) OR (TI (tele# N2 (medicine OR nursing OR health OR consult* OR care OR intervention#)) OR AB (tele# N2 (medicine OR nursing OR health OR consult* OR care OR intervention#))) OR (TI ((consult* OR care) N2 (virtual* OR ehealth OR remote OR video OR electronic)) OR AB ((consult* OR care) N2 (virtual* OR ehealth OR remote OR video OR electronic)))) AND ((TI (physician# OR nurse# OR nursing OR "health care professional" OR "health care personnel" OR "healthcare worker#" OR "healthcare personnel" OR practitioner# OR doctor# OR clinician# OR provider#) OR AB (physician# OR nurse# OR nursing OR "health care professional" OR "health care personnel" OR "healthcare worker#" OR "healthcare personnel" OR practitioner# OR doctor# OR clinician# OR provider#)) OR (TI (((healthcare# OR care# OR health# OR general OR clinic# OR hospital#) N4 (nurse* OR nursing OR profession* OR practitioner# OR clinician# OR personnel OR worker# OR provider# OR staff OR doctor#))) OR AB (((healthcare# OR care# OR health# OR general OR clinic# OR hospital#) N

MEDLINE & CINAHL (EBSCOhost):

#	Query
S22	S6 AND S10 AND S18 AND S21
S21	S19 OR S20
S20	(MH "Australia+")
S19	TX australia* OR queensland* OR "new south wales" OR "northern territory" OR tasmania OR" australian capital territory" OR victoria# OR "western australia" OR "south australia"
S18	S11 OR S12 OR S13 OR S14 OR S15 OR S16 OR S17
S17	(MH "Theory of Planned Behavior") OR (MH "Behaviorism") OR (MH "Decision Making") OR (MH "Thinking"+) OR (MH "Attitude of Health Personnel+") OR (MH "Attitude to Computers")
S16	TI (perceive# OR perception* OR personal OR personalit* OR psychologic* OR reluctan* OR resistance OR self*effic* OR "self*effic*" OR "self effic*" OR "techno*phob*" OR thinking OR think OR thought# OR trust OR view# OR willing OR worry OR worries) OR AB (perceive# OR perception* OR personal OR personalit* OR psychologic* OR reluctan* OR resistance OR "self*effic*" OR "self effic*" OR "techno*phob*" OR "techno phob*" OR thinking OR think OR thought# OR trust OR view# OR willing OR worry OR worries)
S15	TI ((patient# OR client#) N2 cent*d) OR AB ((patient# OR client#) N2 cent*d)
S14	TI ((past OR previous) N2 (experience# OR behavi#r*)) OR AB ((past OR previous) N2 (experience# OR behavi#r*))
S13	TI (emotion* OR confidence OR confident* OR enjoy* OR fear* OR feel* OR identity OR identities OR inertia OR intent* OR interest# OR intrapersonal OR intrinsic OR motivat* OR opinion#) OR AB (emotion* OR confidence OR confident* OR enjoy* OR fear* OR feel* OR identity OR identities OR inertia OR intent* OR interest# OR intrapersonal OR intrinsic OR motivat* OR opinion#)

S12	TI (decision# N2 (making OR made OR make#)) OR AB (decision# N2 (making OR made OR make#))
S11	TI (affect* OR anxiety OR anxieties OR attitude# OR aversion# OR belief# OR bias OR cognitive OR concern# OR behavio#r*) OR AB (affect* OR anxiety OR anxieties OR attitude# OR aversion# OR belief# OR bias OR cognitive OR concern# OR behavio#r*)
S10	S7 OR S8 OR S9
S9	(MH "Health Personnel+")
S8	TI (((healthcare# OR care# OR health# OR general OR clinic# OR hospital#) N4 (nurse* OR nursing OR profession* OR practitioner# OR clinician# OR personnel OR worker# OR provider# OR staff OR doctor#))) OR AB (((healthcare# OR care# OR health# OR general OR clinic# OR hospital#) N4 (nurse* OR nursing OR profession* OR practitioner# OR clinician# OR personnel OR worker# OR provider# OR staff OR doctor#)))
S7	TI (physician# OR nurse# OR nursing OR "health care professional" OR "health care personnel" OR "healthcare worker#" OR "healthcare personnel" OR practitioner# OR doctor# OR clinician# OR provider#) OR AB (physician# OR nurse# OR nursing OR "health care professional" OR "health care personnel" OR "healthcare worker#" OR "healthcare personnel" OR practitioner# OR doctor# OR clinician# OR provider#)
S6	S1 OR S2 OR S3 OR S4 OR S5
S5	(MH "Telemedicine+") OR (MH "Videoconferencing") OR (MH "Remote Consultation+")
S4	TI ((consult* OR care) N2 (virtual* OR ehealth OR remote OR video OR electronic)) OR AB ((consult* OR care) N2 (virtual* OR ehealth OR remote OR video OR electronic))
S3	TI (tele# N2 (medicine OR nursing OR health OR consult* OR care OR intervention#)) OR AB (tele# N2 (medicine OR nursing OR health OR consult* OR care OR intervention#))
S2	TI ((video N1 conferenc*) OR "video*conferenc*") OR AB ((video N1 conferenc*) OR "video*conferenc*")
S1	TI ("tele*health" OR "tele*medicine" OR "tele*nursing" OR "tele*care" OR "tele*consult*" OR "tele*intervention#") OR AB ("tele*health" OR "tele*medicine" OR "tele*nursing" OR "tele*care" OR "tele*consult*" OR "tele*intervention#") OR SO ("tele*health" OR "tele*medicine" OR "tele*nursing" OR "tele*care")
